# Targeting Mitochondrial Network Architecture in Down Syndrome and Aging

**DOI:** 10.3390/ijms21093134

**Published:** 2020-04-29

**Authors:** Nunzia Mollo, Rita Cicatiello, Miriam Aurilia, Roberta Scognamiglio, Rita Genesio, Maria Charalambous, Simona Paladino, Anna Conti, Lucio Nitsch, Antonella Izzo

**Affiliations:** 1 Department of Molecular Medicine and Medical Biotechnology, University of Naples Federico II, 80131 Naples, Italy; 2 Institute of Experimental Endocrinology and Oncology “G. Salvatore”, National Research Council, 80131 Naples, Italy

**Keywords:** Down syndrome, aging, mitochondrial dynamics, mitochondrial network, mitochondrial function, PGC-1α/PPARGC1A, mTOR

## Abstract

Mitochondria are organelles that mainly control energy conversion in the cell. In addition, they also participate in many relevant activities, such as the regulation of apoptosis and calcium levels, and other metabolic tasks, all closely linked to cell viability. Functionality of mitochondria appears to depend upon their network architecture that may dynamically pass from an interconnected structure with long tubular units, to a fragmented one with short separate fragments. A decline in mitochondrial quality, which presents itself as an altered structural organization and a function of mitochondria, has been observed in Down syndrome (DS), as well as in aging and in age-related pathologies. This review provides a basic overview of mitochondrial dynamics, from fission/fusion mechanisms to mitochondrial homeostasis. Molecular mechanisms determining the disruption of the mitochondrial phenotype in DS and aging are discussed. The impaired activity of the transcriptional co-activator PGC-1α/PPARGC1A and the hyperactivation of the mammalian target of rapamycin (mTOR) kinase are emerging as molecular underlying causes of these mitochondrial alterations. It is, therefore, likely that either stimulating the PGC-1α activity or inhibiting mTOR signaling could reverse mitochondrial dysfunction. Evidence is summarized suggesting that drugs targeting either these pathways or other factors affecting the mitochondrial network may represent therapeutic approaches to improve and/or prevent the effects of altered mitochondrial function. Overall, from all these studies it emerges that the implementation of such strategies may exert protective effects in DS and age-related diseases.

## 1. Introduction

Mitochondria are highly dynamic organelles whose function is pivotal for the maintenance of cellular homeostasis [1]. They play an essential role in energy conversion, but also exert important functions in regulating apoptosis and calcium signaling, and support other vital tasks [2,3,4,5]. Mitochondria undergo morphological adjustments/rearrangements driven by dynamic events that vary across cell types and tissues, and in response to external clues. These processes, defined overall as mitochondrial dynamics, regulate the strategic positioning of mitochondria within the cytoplasm and facilitate inter-organelle cross-talk, metabolite transfer, biogenesis and selective mitochondrial autophagy (mitophagy) [6].

Several studies link mitochondrial network architecture to the balance between energy demand and nutrient supply [7]. Indeed, mitochondrial fusion generates tightly configured cristae, which results in closely associated respiratory complexes promoting oxidative phosphorylation [8]. Furthermore, it has been proven that mitochondrial tubulation, upon nutrient deprivation, protects mitochondria from degradation through autophagosomes, allowing them to maximize energy production and provide autophagosomal membranes during starvation [9].

Interestingly, mitochondrial remodeling is a primary signal that shapes the metabolic response during cellular reprogramming and/or quiescence [5]. Mitochondrial dynamics can be involved in the immune response, e.g., controlling the anti-tumor immune response, thus representing a potential target for cancer immunotherapy [10]. Furthermore, Mitra et al. reported a relationship between mitochondrial shape and cell cycle control. A hyperfused mitochondrial state leads to the buildup of cyclin E and entry into S phase [11].

Mitochondrial network architecture is relevant because the organelles are not floating in the cytosol, but rather are held in an organized dynamic interplay through membrane contact sites [12]. Mitochondrial inner and outer membranes come together at the cristae junctions, where mitochondrial contact sites and the cristae organizing system act as a membrane shaping and connecting scaffold. This peculiar architecture has a pivotal importance for multiple mitochondrial functions and for the interactions with other sites [13]. For instance, Dolman et al. found a peri-granular mitochondrial belt positioned predominantly at the basolateral pole of airway epithelia [14]. This belt creates restricted Ca²^+^ domains [15] and a delay in nuclear Ca²^+^ entry, possibly playing an important role in the generation of ATP [16]. Indeed, disturbances in calcium signaling have been implicated in brain aging, and in the pathogenesis of various chronic neurodegenerative disorders [17,18]. Furthermore, mitochondria and endoplasmic reticulum (ER) exhibit tightly coupled dynamics and have extensive contacts. ER tubules play an active role in defining the position of mitochondrial division sites [19]. ER-derived mitochondria-associated membranes (MAMs) are indispensable for mitochondrial dynamics and function. Zhou et al. have recently shown that, when the MAM protein SigmaR1 is deleted in *Sel1L*−/− cells, the ER-mitochondria contacts were reduced, and the mitochondrial dynamics and morphology were rescued [20]. Growing evidence supports the idea that the molecular interactions occurring between mitochondrial membranes and ER might play a crucial role in aging and in age-related diseases [21]. It is interesting to note that Shai et al. have highlighted a little-studied, although highly disease-relevant, contact: the inter-organelle communication between mitochondria and peroxisomes, which plays a physiological function in fatty acid metabolism [22].

Down syndrome (DS) and aging share a perturbation in mitochondrial functionality, manifested as a decline of mitochondrial biogenesis and turnover [23,24,25,26]. A relevant fragmentation of the mitochondrial network [24] and an accumulation of damaged mitochondria [27] have been observed in trisomic cells. During aging, the loss of equilibrium between biogenesis and turnover leads to the accumulation of mutations in mitochondrial DNA (mtDNA) and causes an increased number of damaged mitochondria [28].

In this review, we first discuss the molecular mechanisms involved in mitochondrial network organization and homeostasis, and the effects of their dysregulation. We describe the mitochondrial phenotype in DS and aging, two conditions that share common molecular alterations. We then report evidence indicating that the altered mitochondrial network architecture is possibly responsible for the changes in mitochondrial homeostasis observed in these two conditions. We finally focus on drugs and compounds that have an impact on the architecture of mitochondrial network, and may provide the basis for therapeutic approaches in DS as well as in age-related diseases.

## 2. Mitochondrial Network Architecture

**Figure 1:**
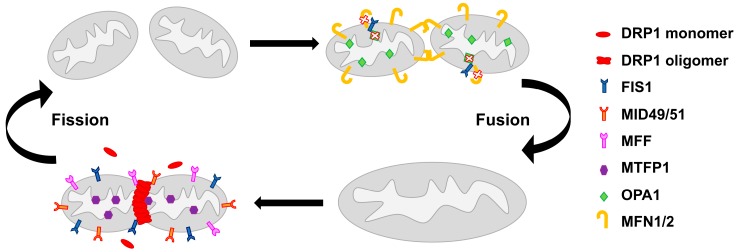
The molecular machinery of mitochondrial network regulation. On the left, the mitochondrial fission process in which several proteins are involved. Dynamin-related protein 1 (DRP1), localized in the cytosol, is recruited by fission 1 protein (FIS1), MID49/51, mitochondrial fission factor (MFF) and mitochondrial fission process protein 1 (MTFP1) to the mitochondrial surface, where it forms a membrane constriction ring. On the right, the mitochondrial fusion process that involves optic atrophy gene 1 (OPA1), which mediates fusion of the inner mitochondrial membrane, and mitofusin 1/2 (MFN1/2), which enhance the fusion of the outer mitochondrial membrane. In mammals, FIS1 can act as a fusion machinery inhibitor by binding to OPA1 and MFN1/2 and blocking their activity.

The multiple functions of mitochondria are mechanistically linked to their morphology, which is defined by ongoing events of fission and fusion of the outer and inner membranes [29]. The dynamic balance of mitochondrial fission and fusion defines patterns of shapes from interconnected networks to fragmented units [30]. These processes are governed by a complex molecular machinery and finely tuned by regulatory proteins [31] (Figure 1).

The dynamin-related protein 1 (DRP1) plays a central role in mitochondrial fission [32], possibly interacting with fission 1 protein (FIS1), an outer mitochondrial membrane protein [30,33]. Mutations or loss of the fission proteins block mitochondrial division, shifting the balance towards fusion [32,34]. DRP1 phosphorylation on serine-616 has been described to induce the translocation of DRP1 to mitochondria, promoting their fission [35]. DRP1 action at mitochondria is affected by MID49 and MID51, two mediators of mitochondrial division of 49 and 51 kDa respectively. MID49/51 are anchored to the mitochondrial outer membrane, where they form foci and rings around mitochondria and directly recruit DRP1 to the mitochondrial surface. Their knockdown reduces DRP1 association, leading to unopposed fusion [36]. The mitochondrial fission factor (MFF) is another essential factor in the mitochondrial recruitment of DRP1 [37]. DRP1 also controls effective T-cell immunosurveillance by regulating cell migration, proliferation, and metabolic reprogramming [38]. This function may account for the role of mitochondrial dynamics in the antitumor immune response. Another fission-relevant protein is the mitochondrial fission process protein 1 (MTFP1), also called MTP18, a mitochondrial inner membrane protein [39]. Its loss results in a hyperfused mitochondrial reticulum, whereas its overexpression increases fragmentation [39,40].

Mitochondrial fusion is controlled by three GTPases, namely the inner membrane protein optic atrophy gene 1 (OPA1) and the outer membrane proteins mitofusin 1 (MFN1) and mitofusin 2 (MFN2) [31]. OPA1 has two proteolytic cleavage sites recognized by two membrane-bound metalloproteases, OMA1 [41] and YME1L [42]. Presenilin-associated rhomboid-like (PARL) protein, a mammalian mitochondrial rhomboid protease, plays a crucial role in proteolytic processing of OPA1, which generates an OPA1 form relevant for cristae maintenance [43]. It should be noted that the oligomerization of OPA1 controls cristae remodeling during apoptosis [43,44]. Low ATP concentrations inhibit the cleavage of OPA1 resulting in mitochondrial fusion, while high ATP levels induce cleavage into a shorter isoform, which supports fission [45,46]. Short forms of OPA1 are involved in mitochondrial fragmentation [45]. The MFN1 protein is involved in apoptosis-associated changes in mitochondrial morphology and function by regulating the activation of BAX on the outer mitochondrial membrane [47]. Mitofusins are negatively regulated by different E3-ubiquitin ligases, miRNAs, oxidative stress and mitophagy [48,49,50,51]. MFN2 is involved in the association between ER-mitochondria contacts and mitochondrial dynamics, as MAM proteins may regulate mitochondrial dynamics by promoting MFN2 oligomerization [20]. Eura et al. identified a mitofusin-binding protein (MIB), which interacts with mitofusin proteins. MIB is essential for cellular functions and negatively regulates the fusion of the mitochondrial membrane by modulating MFN1 function [52]. Individual knockout of any of these genes is lethal in mice, demonstrating that they are essential during embryonic development [53,54].

Interestingly, a recent study highlighted that FIS1 may also act by binding to, and inhibiting, the fusion machinery [55]. This suggests a more complex scenario, in which fusion and fission molecular machineries are strictly interdependent.

It is noteworthy that the fission process, followed by selective fusion, can segregate the dysfunctional mitochondria, and allows their removal by autophagy [56]. This is a form of selective autophagy, the mitophagy, which requires the same protein complexes employed in autophagy to form and clear autophagosomes [57]. Inhibition of fission, by inactivating DRP1, prevents mitophagy in cardiomyocytes [58].

## 3. Mitochondrial Homeostasis

**Figure 2:**
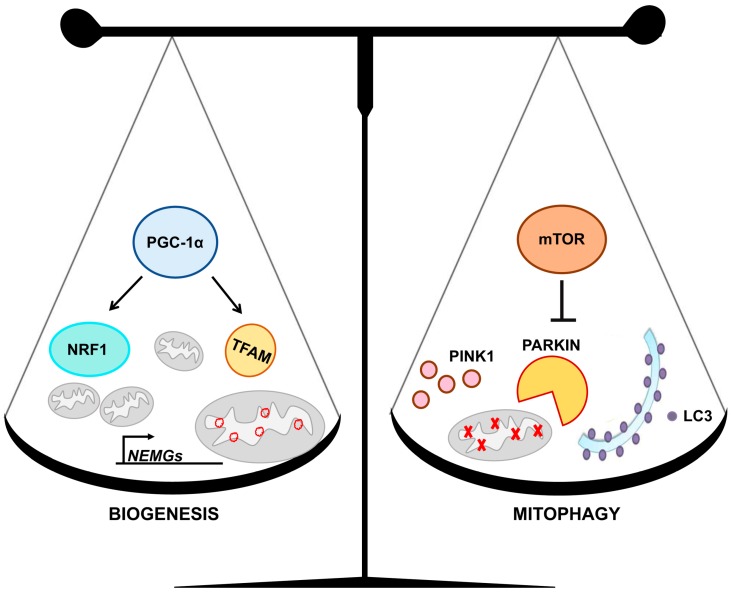
Mitochondrial homeostasis is based on the balanced interplay between biogenesis and mitophagy. On the left, some of the molecular mechanisms of mitochondrial biogenesis are represented. The peroxisome-proliferator-activated receptor γ co-activator-1α (PGC-1α) induces the expression of nuclear respiratory factor 1 (NRF1), which regulates most of the nuclear encoded mitochondrial genes (NEMGs), and transcription factor A (TFAM), which governs mitochondrial DNA (mtDNA). On the right, some aspects of the mitophagy process are represented. Mammalian target of rapamycin (mTOR) signaling negatively regulates the clearance of damaged mitochondria through the pathway dependent upon the PTEN-induced putative kinase 1 (PINK1) and the parkin RBR E3 ubiquitin protein ligase (PARKIN). LC3 promotes autophagosomes formation.

Mitochondrial homeostasis is based on the interplay between mitochondrial biogenesis and mitophagy, two processes that regulate intracellular mitochondrial content and organization [59] (Figure 2).

Mitochondrial biogenesis is a dynamic process that can determine variations in number, size and mass of mitochondria [60]. The peroxisome-proliferator-activated receptor γ co-activator-1α (PGC-1α/PPARGC1A) is a nodal regulator of this process [61]. It is a co-transcriptional regulatory factor that induces the synthesis and/or the activity of several transcription factors, including the nuclear respiratory factor 1 (NRF1), which regulates nuclear encoded mitochondrial genes (NEMGs), and the transcription factor A (TFAM) [60,62], which controls mtDNA transcription, replication and repair [63,64]. Overexpression of *PGC-1α* induces a shift of the mitochondrial network towards fusion by inducing *OPA1* and *MFN1* expression, and/or by repressing *DRP1* expression [25,65,66,67]. Furthermore, chromatin immunoprecipitation assay revealed significantly increased binding of PGC-1α to the *Drp1* promoter [65]. A transcriptional relationship between *PGC-1α* expression and *OPA1* gene was demonstrated in the fruit fly *Drosophila melanogaster*, where the *OPA1*-like gene appears to be regulated in a spatio-temporal fashion by the transcription factor/coactivator Erect wing, the *Drosophila* homolog of human *NRF1* [68]. Moreover, PGC-1α controls mitochondrial dynamics by stimulating *MFN1* and *MFN2* gene expression in an *ERRα*-dependent manner [69,70].

**Figure 3:**
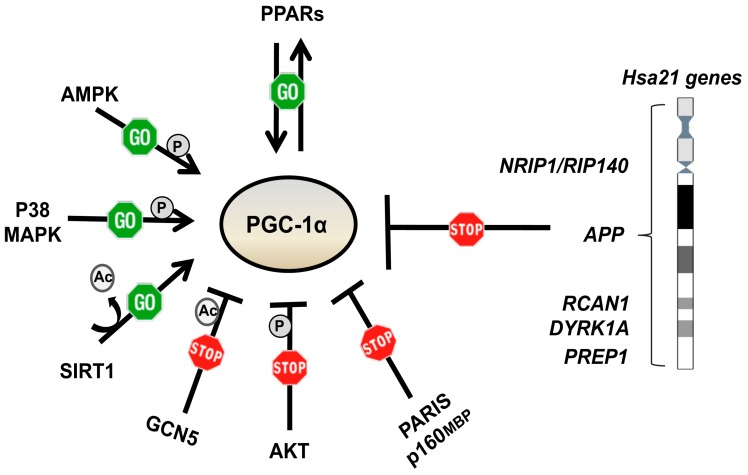
Factors regulating PGC-1α expression and/or activity. The peroxisome-proliferator-activated receptors (PPARs) regulate, and are regulated by, PGC-1α. AMPK, P38 MAPK and SIRT1 positively regulate PGC-1α at post-transcriptional levels. GCN5, AKT, PARIS and p160MBP negatively regulate PGC-1α. PGC-1α expression and/or activity is also regulated by genes mapping to chromosome 21.

PGC-1α is regulated at both the transcriptional and post-translational level (Figure 3).

The peroxisome-proliferator-activated receptors (PPARs) regulate its expression. Both PPAR-α and PPAR-γ are inducers of the *PGC-1α* gene transcription, acting through a PPAR-responsive element in the distal promoter region [71,72]. PGC-1α functions as a potent transcriptional coactivator for PPAR-γ [73]. There is evidence that PGC-1α expression and/or activity is repressed by some chromosome 21 genes, including *NRIP1/RIP140*, *APP*, *DYRK1A*, *PREP1* and *RCAN1* [25], and some miRNAs [74]. Furthermore, PGC-1α is negatively regulated by p160MBP and by PARIS, a KRAB and zinc finger protein that contributes to the neurodegeneration occurring in Parkinson disease (PD) [75,76]. At the post-translational level, AMPK and P38 MAPK activate PGC-1α by phosphorylating threonine-177 and serine-538 for the former [77], and threonine-262, serine-265 and threonine-298 for the latter [78]. AKT, a key component of the insulin signaling pathway, inhibits PGC-1α by phosphorylating serine-570 [79]. In addition, the nutrient sensitive kinase GSK3β phosphorylates PGC-1α for nuclear proteasomal degradation [80]. PGC-1α activity is further regulated by GCN5, through inhibitory acetylation, and by SIRT1, through stimulatory deacetylation [60].

**Figure 4:**
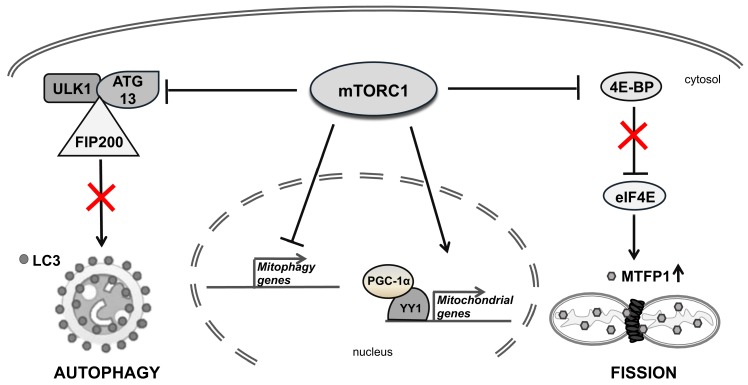
The role of mammalian target of rapamycin complex1 (mTORC1) in regulating mitochondrial homeostasis. mTORC1 inhibits autophagy by phosphorylating the regulatory complex formed by unc-51–like kinase (ULK1) and its interacting proteins, autophagy-related protein 13 (ATG13) and focal adhesion kinase family interacting protein of 200 kDa (FIP200) (on the left). mTORC1 stimulates the mitochondrial fission by phosphorylating 4E-BPs, thus promoting translation initiation of MTFP1 (on the right). mTORC1 also represses mitophagy gene expression and regulates the expression of several mitochondrial genes (in the center).

In addition to this complex regulation, PGC-1α regulates its own transcription via the transcription factor Yin-Yang 1 (YY1), a target of the mammalian target of rapamycin (mTOR) [81]. YY1 binds directly to the promoters of mitochondrial genes, while PGC-1α acts as a transcriptional coactivator of *YY1* in an mTOR-dependent manner. In mammalian cells, PGC-1α interacts with mTOR via the mTOR complex1 (mTORC1), allowing mTOR to control the mitochondrial oxidative function by directly altering the physical interaction between YY1 and PGC-1α [81,82,83]. mTORC1 regulates mitochondrial biogenesis and functions [84,85] by inducing many NEMGs, including components of complexes I and V, mitochondrial ribosomal proteins and TFAM [86]. mTOR controls mitochondrial dynamics by stimulating MTFP1 translation, which affects mitochondrial fission [87] (Figure 4).

In addition to promoting biogenesis, PGC-1α plays a regulatory role in the clearance of damaged mitochondria by affecting their degradation through the mitophagy machinery [57]. The best-characterized pathway that regulate mitophagy is dependent upon the PTEN-induced putative kinase 1 (PINK1) and the parkin RBR E3 ubiquitin protein ligase (PARKIN) [57]. Upon mitochondrial depolarization, PINK1 accumulates on the mitochondrial outer membrane where it is essential to recruit PARKIN to damaged mitochondria [88]. PARKIN is an E3-ubiquitin ligase that enrolls specific autophagic cargo receptors, such as p62, that facilitate sequestration of terminally damaged mitochondria into autophagosomes [89,90]. PARKIN promotes the ubiquitination of the mitofusins in damaged mitochondria both in human dopaminergic neurons and in *Drosophila* [91,92].

PGC-1α mediates the crosstalk between the two opposite processes of biogenesis and mitophagy that result in a precise mitochondrial homeostasis. *Pgc-1α* activation induces lysosome and autophagosome formation, possibly through the upregulation of the transcription factor EB (*Tfeb*) [93], a master regulator of these compartments [94]. Interestingly, PARKIN functionally interacts with PGC-1α to govern mitochondrial homeostasis in dopaminergic neurons. The co-expression of *Pgc-1α* and *Parkin* increases the number of mitochondria, enhances maximal respiration, and accelerates the recovery of the mitochondrial membrane potential [95]. Conditional knockout of *Pgc-1α* leads to loss of dopaminergic neurons [96], while the conditional knockout of *Parkin* leads to the progressive loss of dopaminergic neurons in a *Paris*-dependent manner. On the other hand, *Paris* overexpression leads to the selective loss of neurons in the substantia nigra, which is reversed by its co-expression with either *Parkin* or *Pgc-1α* [76].

Complex interactions are involved in regulating mitophagy. mTORC1 has an established role in inhibiting autophagy by phosphorylating the autophagy regulatory complex formed by unc-51–like kinase (ULK1) and its interacting proteins: the autophagy-related protein 13 (ATG13) and the focal adhesion kinase family interacting protein of 200 kDa (FIP200) [97]. mTORC1 signaling regulates two synergistic processes required for the clearance of damaged mitochondria: (i) general autophagy initiation and (ii) PINK1/PARKIN-mediated selective targeting of uncoupled mitochondria to the autophagic machinery [98] (Figure 4).

## 4. Accelerated Aging in Down Syndrome

Adults with DS experience a process of accelerated aging. Life expectancy of DS subjects is approximately 50–61 years, even though it is notably increasing in developed countries with peaks up to 70 years [99,100]. Clinical manifestations of accelerated aging include muscle hypotonia, osteoporosis [101], premature skin wrinkling, visual and hearing impairment [102], thyroid disorders [103], early menopause and diabetes [104]. The immune function also declines in DS individuals who display a decreased number of T and B lymphocytes, and an increased risk of autoimmune disorders [105]. In many cases, these pathologies occur together [102,106]. In addition to representing the most frequent cause of intellectual disability [107], DS entails an increased risk of developing Alzheimer disease (AD) [108]. Clinical signs of AD are observed in 75% of DS individuals starting from 40 years of age [109,110]. The form and distribution of senile plaques and neurofibrillary tangles, as well as atrophy of neuronal systems, are qualitatively similar to those observed in non-DS AD [111].

The premature onset of aging in DS suggests that trisomy of chromosome 21 increases the biological age of tissues, including the nervous and immune systems. Various aging biomarkers, such as N-glycans of plasma proteins and telomere length, were found altered in DS. Accelerated aging was revealed in DS when plasma N-glycome was evaluated [112]. Furthermore, DS subjects exhibited shorter telomere length, another known marker of aging [113], when compared with control subjects [114,115,116]. The accelerated aging phenotype of DS is mirrored by epigenetic alterations [117]. Epigenome-wide analyses have identified DNA methylation signatures in brain, blood, epithelial cells and extra-embryonic tissues from DS patients [118,119,120,121]. Age acceleration in DS is ranging from 2.8 years in buccal cells to 11.5 years in brain [117]. Magnetic resonance imaging showed a brain predicted age difference higher than 7 years when compared with age-matched controls [122].

## 5. Mitochondrial Homeostasis Is Altered in DS and Aging

**Figure 5:**
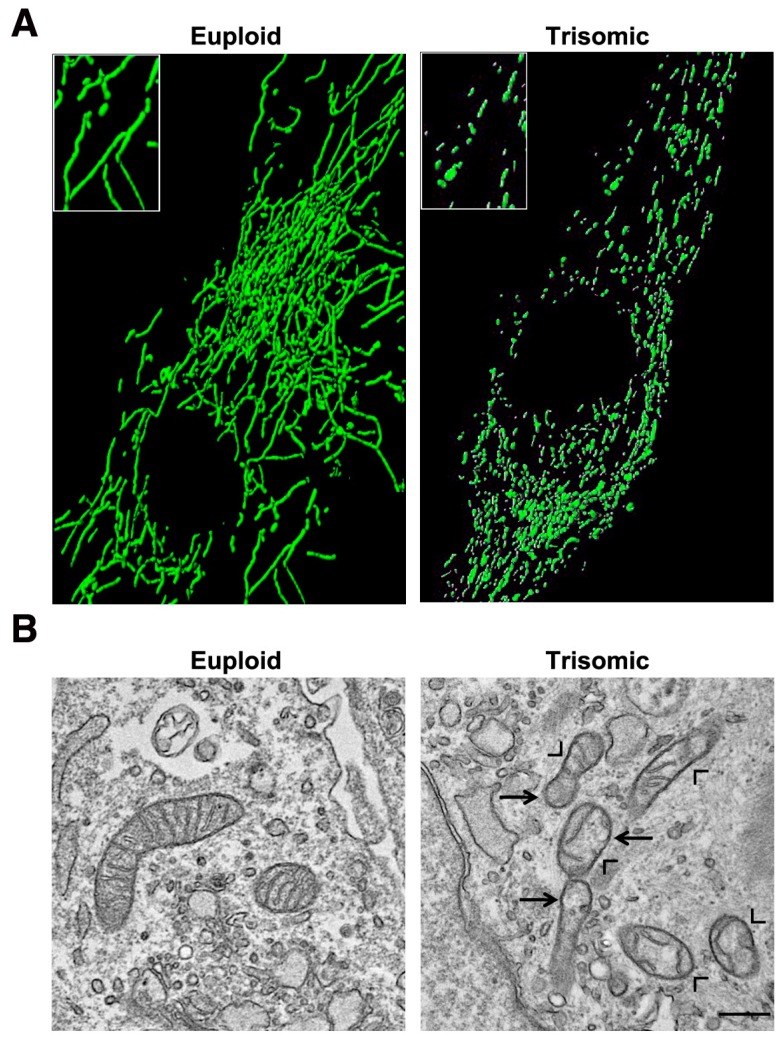
Mitochondrial network and ultrastructure are altered in cells with trisomy of chromosome 21. (**A**) Representative fluorescence microscopy images showing that the mitochondrial network is fragmented in trisomic cells, which display shorter mitochondria instead of branched elongated tubular ones (higher magnification pictures in the inset). Mitochondria were labelled with a mitochondria-targeted green fluorescent protein. (**B**) Representative electron microscopy images showing that mitochondria of trisomic cells are damaged and are characterized by a range of alterations: broken, shorter and less numerous cristae (arrow heads). Moreover, giant swollen mitochondria are also observed in trisomic cells (arrows). Scale bar represents 1 μm.

Many genes and miRNAs mapping to chromosome 21, and upregulated in trisomic cells and tissues, are indirectly involved in mitochondrial function and morphology [25]. It is, therefore, not surprising that a mitochondrial abnormal activity has been documented in DS, both in human subjects and in animal models. In different systems, a significant decrease in respiratory capacity, mitochondrial membrane potential and ATP production, as well as an increase of oxidative stress, have been demonstrated, together with a global disruption of the network organization [25,123,124,125,126]. Mitochondrial alterations in DS have been attributed to a decrease in the abundance and/or activity of PGC-1α [127,128,129]. *PGC-1α* is indeed downregulated in DS fibroblasts [23,130] and in Ts65Dn, a mouse model of DS [131]. Its transcriptional partners, and most of the NEMGs, are accordingly downregulated [23,132]. Izzo et al. demonstrated that the silencing of *NRIP1/RIP140*, a *PGC-1α* repressor, increases *PGC-1α* expression and counteracts mitochondrial dysfunction in DS cells [129]. The mitochondrial network of DS human fibroblasts appeared highly fragmented with an increased number of shorter mitochondria and a smaller average mitochondrial volume [24] (Figure 5A). The disruption of mitochondrial network has been also reported in trisomic mouse embryonic fibroblasts (MEFs) [133], in astrocytes and neurons [125]. In association with an increase of mitochondrial fragmentation in DS, the expression of the two fusion-inducing genes, *OPA1* and *MFN2*, was decreased [24,134], while the expression of *DRP1*, in a different cell model, was increased [134]. Electron microscopy data of trisomic fibroblasts (Figure 5B) and neurons revealed a significant number of damaged mitochondria [23,24,125], with broken, shorter, concentric or highly swollen cristae [23,24]. This is in agreement with the decreased *OPA1* expression in DS [24] and its role in cristae remodeling [44,135].

Many studies in cells and tissues of DS patients, and in DS mouse models, reported aberrant hyperactivation of the AKT/mTOR signaling pathway [136,137,138,139], suggesting that imbalance in autophagy flux regulation in DS leads to negative effects on mitochondrial turnover. A deficient mitophagy process could explain the accumulation of damaged mitochondria in DS. Indeed, Bordi et al. recently demonstrated an overall significant downregulation of transcriptional factors essential to support mitophagy activation in DS fibroblasts. Two alterations in mitophagy pathways were evidenced in DS cells: the downregulation of *PARKIN*, associated with decreased PINK1 dependent mitophagy signaling, and the suppression of mTOR-mediated autophagy [27]. The sustained activation of the mTOR pathways is observed in DS, as well as in neurodegenerative diseases, including AD [140].

A decline in the efficiency of mitochondria in generating energy and consuming oxygen also occurs during aging [141]. Mitochondrial morphological alterations, similar to those found in DS, are observed in aging and age-related diseases [23,24,142]. With increasing age, *C. elegans* neurons and muscles show an increase of mitochondrial fragmentation associated with a decrease in mitochondrial volume [143,144]. Mitochondria of old endothelial cells show a significant perturbation of the fission/fusion machinery [145]. Ron-Harel et al. observed a decrease in the number and activation of naïve T cells isolated from aged mice. While young T cells showed robust mitochondrial biogenesis and respiration upon activation, aged T cells displayed smaller mitochondria with lower respiratory capacity [146].

PGC-1α has emerged as an important player in aging. Its expression in skeletal muscle is decreased with aging in both rodents and humans [147,148]. It extends the health span and life span of a mouse model of premature aging arising from mitochondrial defects [149]. Moreover, deletion of *NRIP1/RIP140* extends mice longevity, increases autophagy and delays cell senescence [150].

Formation and processing of autophagosomes decrease with aging, which may lead to a deficit of mitophagy [151]. In age-related pulmonary fibrosis, defects in mitophagy and mitochondrial biogenesis have been implicated in both cellular apoptosis and senescence during tissue repair [152]. *PARKIN* overexpression in aging models affects mitochondrial dynamics and extends lifespan [153].

mTOR has been implicated in mitophagy, as well as in many of the processes that are associated with aging, including cellular senescence, immune response, stem cell regulation, autophagy and mitochondrial function [98,154]. It is well accepted that the downregulation of the mTOR signaling pathway is a central regulatory process of pro-longevity in mammals [155,156]. The downregulation of this pathway produces an extension of lifespan in multiple organisms [157].

## 6. Pharmacological Strategies to Target Mitochondrial Network Architecture

The literature data demonstrate that the molecular mechanisms responsible for altered mitochondrial network architecture in DS and aging have been at least in part identified. By targeting these mechanisms, the correct architecture can be possibly restored, thus promoting the recovery of the mitochondrial quality and function. This recovery is likely to induce an improvement of DS and age-related diseases and possibly an extension of the lifespan.

### 6.1. Metformin

Since PGC-1α is the main actor in regulating mitochondrial function and network organization, drugs which enhance its expression and/or activity would improve the mitochondrial phenotype. These drugs can be appropriately proposed as a therapeutic approach to fight against DS and age-related diseases.

**Figure 6:**
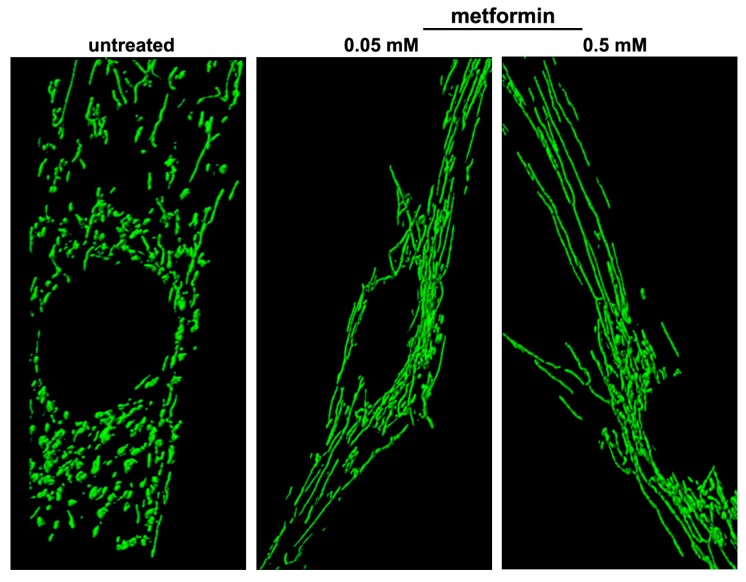
Metformin counteracts mitochondrial network fragmentation in cells with trisomy of chromosome 21. Representative fluorescence microscopy images showing a branched and elongated tubular morphology of the mitochondrial network in trisomic cells treated with metformin at two different concentrations. Mitochondria were labelled with a mitochondria-targeted green fluorescent protein.

One of these drugs, the biguanide metformin, induces PGC-1α activity through AMPK activation. It promoted mitochondrial biogenesis and reversed the mitochondrial network fragmentation in DS cells, inducing a branched and elongated tubular morphology of the network (Figure 6) [25]. Concomitantly, mitochondrial cristae remodeling occurred in metformin-treated cells and the number of damaged mitochondria was significantly decreased [25]. Together with the rescue of mitochondrial network architecture, the expression of genes of the fusion machinery, namely *OPA1* and *MFN2*, was increased. All these events were associated with an increase in oxygen consumption and ATP production, and a decrease in the generation of reactive oxygen species (ROS) [25].

The mechanism by which metformin activates AMPK is not clearly established. Zhou et al. proposed that it might stimulate AMPK phosphorylation by upstream kinase(s), or inhibit its dephosphorylation by protein phosphatase(s) [158]. The most popular hypothesis is that metformin can act as an inhibitor of complex I of the respiratory chain [159]. This inhibition may have multiple downstream effects but, importantly, it would lead to a change in the AMP/ATP ratio, which then activates AMPK. However, there are evidences that AMPK can be activated by mechanisms other than changes in the cellular AMP-to-ATP ratio [160].

**Figure 7:**
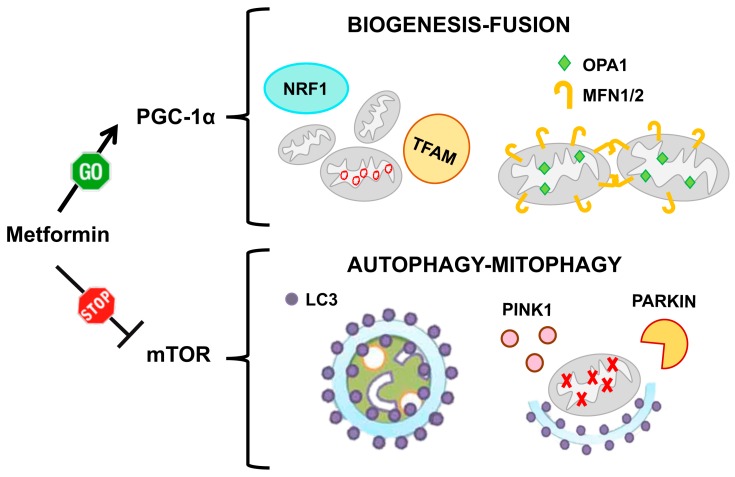
Metformin action on mitochondrial homeostasis. Metformin acts on mitochondrial homeostasis by two pathways: on one side it increases mitochondrial biogenesis and induces mitochondrial fusion via PGC-1α activation. On the other side it induces the formation of autophagosomes and promotes the clearance of damaged mitochondria via mTOR inhibition.

In addition to its role as PGC-1α activator, metformin inhibits, both in vitro and in vivo, mTORC1 signaling (Figure 7), either via AMPK-dependent [161] or via AMPK-independent mechanisms [162]. AMPK itself induces autophagy by directly phosphorylating ULK1, a key initiator of the autophagic process [163]. ULK1 regulates mitophagy through the interaction with the FUNDC1 protein (localized in the mitochondrial membrane), and promotes the translocation of damaged mitochondria to autophagosomes [164]. All these mechanisms might account for the ability of metformin to induce mitophagosome formation and to promote autophagic degradation of dysfunctional mitochondria. Consistently, in metformin-treated cultures of peripheral blood mononuclear cells (PBMCs) from healthy subjects, the mRNA expression of mitophagy-related genes, including *PINK1* and *PARKIN*, was increased and overall protein expression of many mitophagy markers was upregulated [165]. There is evidence that PBMCs from patients with type 2 diabetes mellitus (T2DM) exhibit attenuated mitophagy and altered mitochondrial morphology and function. Interestingly, patients with T2DM displayed augmented mitophagy in their mononuclear cells after receiving metformin monotherapy for 3 months [166].

Many efforts have been made in order to delay the aging process and to counteract age-related disorders by pharmacological approaches. Due to its properties, metformin has been widely investigated as anti-aging agent. It increases lifespan in mouse [167] and *C. elegans* [168], even though a similar effect was not observed either in *Drosophila* or in rats [169]. These results suggest that metformin might modulate fundamental pathways that underlie aging processes and multiple age-related conditions in humans. For this reason, it is not surprising that many clinical trials are in progress to investigate the effects of metformin in aged people and/or on aging processes.

“Targeting Aging with Metformin” (TAME) was one of the first large clinical trial projects to be proposed, in 2016 [170] although as of today, March 2020, it is not yet reported by ClinicalTrials.gov. This trial aimed to study the effects of metformin on occurrence of a composite outcome that includes cardiovascular events, cancer, dementia, and mortality. Three thousand human subjects, aged between 65 and 79 years, would be recruited and treated for 4 years with 1500 mg of metformin per day or placebo. Rather than merely investigating the effects on extended lifespan, the ‘*primum movens*’ of the trial is to investigate whether chronic metformin administration can promote healthy aging.

The other four completed and six ongoing trials are summarized in Table 1.

### 6.2. Other PGC-1α Activating and/or mTOR Inhibiting Drugs

The AMPK synthetic agonist AICAR (5-aminoimidazole-4-carboxamide-1-D-ribofuranoside) is an adenosine analog, which is carried into the cells by adenosine transporters and directly activates AMPK [57]. As described for metformin, AMPK activation induces PGC-1α activity. Moreover, AMPK directly phosphorylates at least two proteins to induce rapid inhibition of mTORC1 activity: the TSC2 tumor suppressor and the critical mTORC1 binding subunit raptor [176]. AICAR was tested in aged animals and in aging models. In immortalized MEFs, which were modified to obtain an aging model, AMPK activation by AICAR was associated with mitochondrial fusion and the autophagy/mitophagy pathway was switched on [177]. AICAR treatment increased proliferation, attenuated senescence-associated changes in mesenchymal stromal cells [178] and reduced interstitial fibrosis in aging mice [179]. AICAR treatment of aged mice promoted the recovery of motor and memory function, possibly by increasing the expression of mitochondrial genes in the muscle and neural plasticity in the hippocampus [180].

The PPAR agonist pioglitazone, a member of the thiazolinedione family, induces PGC-1α by activating the PPAR-γ signaling. It protects mitochondria from injury-induced mitochondrial dysfunction preserving ATP production [181]. In DS fibroblasts, pioglitazone modulated the mitochondrial network architecture [67]. It induced the expression of the fusion genes *OPA1* and *MFN1*, while repressing that of the fission gene *DRP1*, and increasing the mRNA and protein levels of PGC-1α. A significant increase of the ATP content and oxygen consumption rate, and a significant decrease of ROS production, provided strong evidence of an overall improvement of mitochondrial bioenergetics in trisomic cells [67]. Pioglitazone was also tested as an anti-aging agent. It exhibited anti-aging properties in *Drosophila* [182] and attenuated aging-related disorders in aged apolipoprotein E deficient mice [183]. The activation of PPAR-γ by pioglitazone, in vivo, reduced oxidative stress in aging rat cerebral arteries through the upregulation of UCP2 [184]. Using a clinically-relevant dose in a long-term treatment, pioglitazone was able to blunt several indices of aging, but apparently neither age-related cognitive decline nor peripheral/central age-related increases in inflammatory signaling were reversed [185]. On the other side pioglitazone, at a dosage lower than that used to treat diabetes, ameliorated learning and memory impairment in a mouse model of dementia by increasing *LRP1* expression in the hippocampus [186]. In all these experiments no information about mitochondrial network and function was gained.

Polyphenolic compounds have been described, which are able to induce PGC-1α activity. They have been tested both in DS and aging [66].

Resveratrol (3,40,5-trihydroxy-trans-stilbene) is a natural polyphenolic compound, found in red wine and in the root of some plants, that activates PGC-1α through SIRT1 and AMPK enzymatic activities [187]. In vitro studies indicate that biological effects of resveratrol on cellular senescence or other cellular processes may vary depending on cell types and certain contexts [188,189]. Resveratrol was able to extend lifespan in yeast, worms, flies and mice [190,191,192,193]. Supplementation of this drug for 4 weeks efficiently decreased oxidative stress and ameliorated some age-related diseases in T2DM patients [194,195]. Valenti et al. found that resveratrol enhances mitochondrial functions by upregulating PGC-1α/SIRT1/AMPK axis and promotes the proliferation of neural precursor cells derived from the DS mouse model Ts65Dn [66]. They further demonstrated that resveratrol regulates the mitophagy processes and restores the mitochondrial architecture. In rats and PC12 cells damaged by rotenone, resveratrol pretreatment prevented an OPA1 and MFN2 decrease, increased mitochondrial biogenesis and fusion, mtDNA copy number and mitochondrial mass and protected neuronal cells [196]. In rat hearts, which underwent ischemia-reperfusion injury, resveratrol induced the “SIRT1/SIRT3-FOXO3A-PINK1-PARKIN-mitochondrial fusion/fission-mitophagy” cascade leading to cardioprotection [197]. In addition, resveratrol promoted mitochondrial elongation via DRP1/PARKIN/PINK1, attenuating senescence in cardiomyocytes [198]. Resveratrol induced autophagy by directly inhibiting the mTOR-ULK1 pathway in HeLa cells in an ATP-competitive manner [199]. It also activated autophagy and ameliorated motor impairment in a mouse model of PD [200].

A therapeutic benefit on mitochondrial activity was demonstrated by another polyphenol, the Epigallocatechin-3-gallate (EGCG), in cellular and murine models of DS. Indeed, EGCG treatment renews the capacity of DS cells to produce energy by mitochondrial oxidative phosphorylation system and to prevent mitochondrial ROS overproduction [130]. The treatment with EGCG of neural progenitor cells isolated from the hippocampus of Ts65Dn mouse reactivates mitochondrial bioenergetics and biogenesis, and promotes neural progenitor cell proliferation [66]. Additionally, in this case mitochondrial network is affected as EGCG interacts with OMA1, and potently inhibits its activation to attenuate OPA1 cleavage. This improves mitochondrial dynamics and maintains mitochondrial morphology and cristae structure [201]. Using in vitro and in vivo subarachnoid hemorrhage models, Chen et al. demonstrated that EGCG modulates mitochondrial fragmentation by regulating the expression of *Drp1*, *Fis1*, *Opa1*, *Mfn1* and *Mfn2* [202]. EGCG administration in adult DS patients, in the first randomized controlled clinical trial using a dietary supplement, showed a significant improvement in memory and executive function, and facilitated adaptive behavior [203]. EGCG treatment demonstrated to improve the replicative life span of fibroblasts [204] and to promote healthy lifespan in *C. elegans* [205].

Urolithin A (UA) is a small natural compound prominent in pomegranate. Ryu et al. identified UA as a first-in-class natural compound that induces mitophagy both in vitro and in vivo, via AMPK signaling activation [206,207]. Dietary UA treatment induced mitophagy, prevented the age-related accumulation of dysfunctional mitochondria in *C. elegans* and rodents, and prolonged lifespan in *C. elegans* [206]. Mitochondrial network in *C. elegans* was more fragmented following UA treatment, consistent with the fact that mitophagy and mitochondrial fission are intimately linked. This fragmentation was accompanied by lower mRNA levels of *FZO1* and *OPA1*, two major components of the mitochondrial fusion machinery [206]. Mitophagy enhancement by UA was able to diminish insoluble Aβ40 and Aβ42, and to abolish tau hyperphosphorylation in animal models of AD [208]. UA was tested in a first-in-human anti-aging clinical trial in which it demonstrated a favorable safety profile with a molecular signature of improved mitochondrial activity [209].

### 6.3. mTOR Direct Inhibitors

As mitochondrial architecture is strictly related to the maintenance of homeostasis, mitophagy is a fundamental process to ensure the elimination of damaged mitochondria as well as to counteract excessive fission [210]. On these premises, it is time to introduce a drug which directly suppresses mTOR signaling, the rapamycin, also known as sirolimus.

Rapamycin is an antifungal agent produced by *Streptomyces hygroscopicus*, discovered in soil samples from Rapa Nui Polynesian island. Its use is currently approved to prevent graft rejection and to treat autoimmune disorders. Its discovery opened the way to the identification of its target, the kinase TOR. Rapamycin is a widely used autophagy inducer, which acts through an inhibitory effect on mTOR [211]. Numerous studies have shown that rapamycin and some of its analogues (called rapalogues) extend lifespan in yeasts, worms and flies [212], as well as from 10% to 30% in mice [213,214]. Rapamycin can work as an anti-aging agent for three main reasons: (i) it slows down cell senescence [215]; (ii) it attenuates mitochondrial dysfunction by inducing mitophagy [216]; and (iii) it counteracts fission through inhibition of the mTOR signaling [217]. In addition, it mitigated epigenetic aging in human keratinocytes independently of replicative senescence [218]. The effects of rapamycin were tested in Ts65Dn mice by setting up a new intranasal delivery protocol that maximizes brain delivery and reduces systemic side effects (12 weeks of treatment). This treatment was able to counteract the aberrant signaling of mTOR and of its downstream targets in the hippocampus of Ts65Dn mice [219,220]. Furthermore, rapamycin treatment appeared to correct many of the metabolome changes detected in both young and old Ts65Dn mice [221]. Mitochondrial architecture parameters during rapalog administration were not investigated in aging or DS. However, these drugs may restore the mitochondrial network architecture in both conditions by suppressing the mTORC1 regulation of MTFP1. Indeed, the suppression of mTORC1 activity by pharmacological means, including rapamycin, caused mitochondrial hyperfusion, branching and circularization in MEFs [87].

### 6.4. Drugs Affecting Mitochondrial Fission/Fusion Machinery

The following compounds, which either inhibit fission or promote fusion, have a documented role in modulating the mitochondrial network architecture. It will be of great interest to better investigate their effects in DS and age-related diseases, since excessive mitochondrial fission and decreased fusion are key features of these conditions. It is well known that an increased mitochondrial fusion is essential for longevity in *C. elegans*. Likewise, mitochondrial fusion inhibition reduces its lifespan, even though other factors certainly play a role in this delicate equilibrium [222].

Among the inhibitors of mitochondrial fission, mdivi-1, P110, Dynasore and Drpitor1, which all decrease fragmentation by targeting DRP1, are going to be considered for clinical trials in human diseases, including AD, PD and Huntington disease [223].

Mdivi-1 (mitochondrial division inhibitor 1) is a quinazolinone derivative that attenuates mitochondrial division in yeast and mammalian cells by selectively inhibiting DRP1 [224]. Mdivi-1 decreases DRP1 levels and activity, counteracts mitochondrial fragmentation enhancing fusion and increases the enzymatic activities of complexes I, II and IV [225]. Consistently, Mdivi-1 restored mitochondrial network organization and mitochondrial energy production, and improved proliferation and neuronal differentiation of hippocampal progenitor cells derived from the DS mouse model Ts65Dn [134]. According to in vitro and in vivo studies, Mdivi-1 ameliorates the deficit of mitochondrial dynamics [226,227].

P110 is a synthetic peptide, which selectively inhibits DRP1 enzyme activity blocking DRP1/FIS1 interaction in cultured neurons [228]. In a model of PD, P110 exerted a neuroprotective effect by inhibiting mitochondrial fragmentation and ROS production. It increased neuronal cell viability by reducing apoptosis and autophagic cell death. Minimal effects were observed on mitochondrial fission and cell viability under basal conditions [228]. P110 showed beneficial effects in AD. Treatment with this drug significantly prevented mitochondrial structural and functional deficits, improved behavioral deficits, and reduced Aβ accumulation, energetic failure and oxidative stress in cellular and animal models of AD [229].

Dynasore is a small molecule that interferes in vitro with the GTPase activity of DRP1 [230]. In cultured adult mouse cardiomyocytes subjected to oxidative stress, Dynasore counteracted mitochondrial fission induced by oxidative stress, increased cardiomyocyte survival and viability, and reduced the depletion of cellular ATP [231]. Unfortunately, Dynasore inhibited also Dynamin 1, a protein required for clathrin-mediated endocytosis, and for synaptic vesicle endocytosis in neurons, which could be necessary for memory formation in mice [232].

Drpitor1 is a compound identified as a DRP1 inhibitor after in silico screening [233]. Drpitor1 (and its congener Drpitor1a) inhibited the GTPase activity of DRP1 without inhibiting the GTPase activity of Dynamin 1. These compounds showed greater potency than the current standard DRP1 GTPase inhibitor, Mdivi-1. Drpitor1a prevented mitochondrial fission and ROS production during ischemia-reperfusion injury [233].

Among fusion-promoting compounds, Miret-Casals et al. identified, by high-throughput screening, small molecules that promote mitochondrial elongation in an MFN1/MFN2-dependent manner. One of them, leflunomide, showed the highest activity on the MFN2 promoter [234]. Leflunomide is a drug currently used to treat rheumatoid arthritis [235]. It induced the expression of the mitochondrial fusion proteins MFN1 and MFN2 in C2C12 muscle and in HeLa cells. Furthermore, the expression of short forms of OPA1 were slightly decreased and DRP1 was repressed [234].

Another small molecule, the hydrazone M1, restored the mitochondrial tubular network in response to genetically or chemically induced fragmentation [236]. Administration of M1 significantly promoted mitochondrial fusion in high glucose-treated cardiomyocytes. It consistently prevented mitochondrial fission and enhanced the mitochondrial respiratory capacity [237]. Treatment with M1 promoted mitochondrial fusion also in human induced pluripotent stem cell-derived cells [201]. Buck et al. demonstrated that mitochondrial fusion induced by M1 promotes the generation of memory-like T cells [5].

A summary of all the drugs described is shown in Table 2.

## 7. Conclusions

The amount of evidence that underlines the importance of mitochondrial dynamics in human physiology and pathology is accumulating more and more. The molecular pathways that regulate fission/fusion processes, as well as those, closely related, that govern mitochondrial biogenesis and mitophagy are being further clarified in detail. The picture that emerges is that these pathways are articulated and interconnected, as can be expected for processes that must respond to a wide variety of different stimuli. Two pathways, which play a central role in the control of bioenergetics and nutrient use in cells, emerge as essential regulators of mitochondrial dynamics: the PGC-1α and mTOR pathways. They are closely interconnected and are both involved in the organization of the mitochondrial network architecture. Stimulation of PGC-1α mainly promotes biogenesis and fusion of the mitochondria, while inhibition of mTOR mainly promotes the autophagy and mitophagy processes. What emerges clearly from the scientific literature is that both these pathways are altered in a similar way in the two conditions we have considered here, DS and aging, in which PGC-1α pathway is inhibited, while mTOR signaling is hyperactive. This leads to fragmentation of mitochondrial network and accumulation of damaged mitochondria. If this hypothesis is correct, a drug that activates the PGC-1α pathway and inhibits the mTOR pathway would be considered ideal to fight DS and age-related diseases. A drug with these characteristics exists: it is metformin. The attention of many research groups has been focused on this line, which has led to the initiation of many clinical trials aimed at achieving healthier aging. More recently, metformin also emerged as a drug that could antagonize some of the defects caused by DS, both the once already present at birth and those that occur later in life. In DS, alterations of the mitochondrial dynamics occur early in fetal development, and appear to be a consequence of the genomic imbalance. Early correction of these mitochondrial alterations is likely to improve the phenotype, and clinical trials testing this hypothesis should be performed. In the same way, it seems useful to continue the experimentation of many other drugs that act on the mitochondrial dynamics, with the aim of testing the most promising ones in clinical trials.

Special attention should be paid in these future clinical trials to identify the correct dose, as the dosage of many of these molecules may be critical. Drugs that play a role at low doses may have an opposite role at high doses in promoting the activation or inhibition of other pathways that may be antagonistic. From this perspective, great attention should be paid to the accuracy and personalization of the trials. It would be useful to identify parameters that allow to directly assess, in each individual, the extent to which a specific dose of a particular drug is affecting the pathways that we intend to modulate.

## Figures and Tables

**Table 1:** Clinical trials testing metformin as anti-aging drug, reported by ClinicalTrials.gov, at March 2020.

Title and Date	Objectives	Dose and Outcome Measures
**Effects of metformin on longevity gene expression and inflammation in pre-diabetic individuals.** **June 2010–March 2013**	To study the role of the AMPK pathway on longevity genes and inflammation in pre-diabetic setting. **Completed.**	**500 mg tris in die** Metformin induced changes in the expression of longevity genes *SIRT1*, *p66Shc*, *mTOR*, *p53* in peripheral blood mononuclear cells [171].
**MILES** **Metformin in Longevity Study.** **October 2014–December 2017**	To determine if metformin restores the gene expression profile of old, glucose intolerant adults to that of young healthy subjects. **Completed.**	**1700 mg/day** Metformin regulates metabolic and non-metabolic pathways in skeletal muscle and subcutaneous adipose tissues of older adults [172,173].
**MASTERS** **Metformin to augment resistance and training adaptations in older adults.** **January 2015–June 2018**	To determine whether metformin can enhance the benefits seen during resistance exercise, such as increased muscle mass and strength. **Completed.**	**1700 mg/day** Results do not support the use of metformin to enhance the benefits of physical activity in healthy elderly people [174].
**Phase 1 Study of the effects of combining topical FDA-approved drugs on age-related pathways on the skin of healthy volunteers.** **March 2017–February 2019**	To examine the effects of FDA approved medications, including metformin, on skin aging when applied in topical form. **Completed.**	**Topical metformin applied to the skin.** Primary measure: profile of gene transcript changes. Secondary measure: wrinkle score.
**MATE** **Metformin and Aging Trial in the Elderly: A pilot and feasibility study.** **May 2018–April 2020**	To test whether chronic metformin administration reduces aging-related biochemical parameters and improves physical performance.	**500 mg every 6 to 8 h** Primary measure: frailty measured by the short physical performance battery, a group of measures that combines the results of the gait speed, chair stand and balance tests. Secondary measure: effect of metformin on senescent markers.
**Anti-Aging, Pro-Autophagy effects of Metformin in Adults with Prediabetes.** **September 2017–July 2021**	To demonstrate that metformin therapy increases cellular senescence and autophagy.	**1500 mg/day** Primary measure: change in Leucocyte LC3 Score.
**Metformin for Preventing Frailty in High-risk Older Adults.** **April 2016–October 2022**	To demonstrate that metformin modulates diabetes/insulin resistance and inflammation will prevent and/or ameliorate the progression of frailty.	**1000 mg twice a day** Primary measure: frailty, measured by validated standardized criteria [175].
**Metformin to prevent inactivity-induced loss of muscle health during aging.** **July 2017–July 2022**	To investigate metformin as a preventive strategy to maintain muscle and metabolic health in bed ridden older adults.	**2000 mg/day** Primary measure: change in muscle size from baseline to 5 days of bed rest (determined by magnetic resonance imaging).
**VA-IMPACT.** **Effects of Metformin on Atherosclerotic Cardiovascular Outcomes in Pre-Diabetes.** **February 2019–August 2024**	To demonstrate that metformin reduces the risk of death, heart attacks, and/or strokes in patients who have pre-diabetes and heart or blood vessel problems.	**1000 mg/day–2000 mg/day** Primary measures: death; non-fatal myocardial infarction or stroke; unstable angina with acute myocardial ischemia; or coronary revascularization. Secondary measures: cumulative/recurrent incidence of the primary measures; time to new/recurrent diagnosis of a malignancy; time to new diagnosis of T2DM.
**Does insulin sensitivity impact the potential of metformin to slow aging?** **March 2020–April 2024**	To demonstrate who may benefit from metformin treatment to slow aging.	**1500 mg/day** Primary measures: change in insulin sensitivity (determined by a hyperinsulinemic-euglycemic clamp); evaluation of the mitochondrial function. Secondary measures: 5-day continuous glucose monitoring; change of aging biomarkers in blood.

**Table 2:** Drugs and compounds that target mitochondrial network architecture.

Drug/ Compound	Activity on Mitochondrial Network	Tested in DS	Tested in Aging
**Metformin** (FDA approved)	Induces OPA1 and MFN2 [24] and inhibits DRP1 [238].	In vitro: [24].	In vitro: [239]. Animal models: [167,168]. Human: [169,171,172,173,174,175].
**AICAR**	Induces OPA1 and MFN1 [177].	Not tested	In vitro: [177,178]. Animal models: [179,180].
**Pioglitazone** (FDA approved)	Induces OPA1 and MFN1 and inhibits *DRP1* [67].	In vitro: [67].	Animal models: [182,183,184,185].
**Resveratrol** (nutraceutic)	Inhibits DRP1 [198,238]. Regulates DRP1, FIS1, OPA1, and MFN2 [196].	In vitro: [66].	In vitro: [188,189]. Animal models: [190,191,192,193]. Human: [194,195].
**Epigallocatechin-3-gallate (EGCG)** (nutraceutic)	Regulates *Drp1*, *Fis1*, *Opa1*, *Mfn1*, and *Mfn2* [202].	In vitro: [66,130]. Human: [203].	In vitro: [204]. Animal models: [205].
**Urolithin A (UA)** (nutraceutic)	Inhibits *OPA1* and *FZO1* [206].	Not tested	Human: [209].
**Rapamycin** (FDA approved)	Induces MTFP1 [87].	In vitro: [218]. Animal models: [219,221].	In vitro: [215,240]. Animal models: [213,214,216,241].
**Mdivi-1**	Inhibits DRP1 [134,224].	In vitro: [134].	Not tested
**P110**	Inhibits DRP1 [228].	Not tested	Not tested
**Dynasore**	Inhibits DRP1 [230].	Not tested	Not tested
**Drpitor1**	Inhibits DRP1 GTPase activity [233].	Not tested	Not tested
**Leflunomide** (FDA approved)	Induces MFN1 and MFN2 and inhibits OPA1 short isoform [234].	Not tested	Not tested
**Hydrazone M1**	Induces MFN2 and inhibits DRP1 [236].	Not tested	Not tested
